# Supplementary material for: A set of multi-entry identification keys to African frugivorous flies (Diptera, Tephritidae)
Source: Zookeys. 2014 Jul 24;(428):97–108. doi: 10.3897/zookeys.428.7366 (PMC4143993; doi:10.3897/zookeys.428.7366)
Supplement: Supplementary material 6 — Key to Ceratitis [file zookeys-428-097-s006.zip › SF6_ZooKeys_key to Ceratitis/key/SF6_ZooKeys_key to Ceratitis/Media/Html/Ceratitis serrata .htm]

Ceratitis (Pardalaspis) serrata sp


***Ceratitis*** **(*Pardalaspis*)
*serrata* De Meyer**

*Ceratitis* (*Pardalaspis*)
*serrata* De Meyer, 1996 : 24.

Body
length: 6.5 mm; wing length: 6.0 mm.

Female

Head.
Antennal segments missing. Frons yellow in ground color, weakly silvery in the
middle; with dispersed short hairs, darker than frons. Ocellar triangle dark.
Face yellow-white, no median band. Occiput moderately swollen below, pale
whitish. Chaetotaxy normal for subgenus.

Thorax.
Ground colour of mesonotum with an orange tinge; with darker coloration around
setal sockets and� some darker streaking, not clearly defined. Postpronotum
same colour as mesonotum. Chaetotaxy normal for subgenus. Mesonotum with pale
pilosity. Anepisternum with darker hairs over entire surface. Three
anepisternal bristles.� Scutellum yellow with apical markings black; basally
with two dark spots.

Legs
yellow; setation typical of subgenus. Hind femur with dorsal hairs dark
apically. Setae dark, posterior row on front femur dark.

Wings
with brownish bands, yellow markings strongly reduced. Banding, setation and
venation normal for subgenus. Marginal band continuous; discal band joined with
marginal band; cross-vein r-m at middle of discal cell; vein R_1_
ending before cross-vein r-m.

Abdomen.
Grey-brown, with orange tinge and clearly defined spots. Pattern of spots and
setation normal for subgenus. Oviscape short, shorter than abdominal terga 3-6.

Male

Unknown.

(Description
after De Meyer, 1996)
